# *Toxoplasma gondii* Infection in Patients with Psychiatric Disorders from Western Romania

**DOI:** 10.3390/medicina58020208

**Published:** 2022-01-30

**Authors:** Sebastian Grada, Alin Gabriel Mihu, Cristina Petrescu, Oana Suciu, Iosif Marincu, Maria Alina Lupu, Tudor Rares Olariu

**Affiliations:** 1 Discipline of Parasitology, Department of Infectious Disease, Victor Babes University of Medicine and Pharmacy, 300041 Timisoara, Romania; seba.grada@gmail.com (S.G.); alinmg@yahoo.com (A.G.M.); imarincu@umft.ro (I.M.); 2 Center for Diagnosis and Study of Parasitic Diseases, Victor Babes University of Medicine and Pharmacy, 300041 Timisoara, Romania; 3 Department of Biology and Life Sciences, Vasile Goldis University of Medicine and Pharmacy, 310300 Arad, Romania; 4 Bioclinica Medical Analysis Laboratory, Dreptatii Street, nr. 23, 310300 Arad, Romania; 5 Department of Microbiology, Victor Babes University of Medicine and Pharmacy, 300041 Timisoara, Romania; cpetrescu64@yahoo.com (C.P.); oana_suciu96@yahoo.com (O.S.); 6 Clinical Laboratory, Institute of Cardiovascular Diseases, 300041 Timisoara, Romania; 7 Clinical Laboratory, Municipal Clinical Emergency Teaching Hospital, 300041 Timisoara, Romania

**Keywords:** epidemiology, Western Romania, psychiatric patients, *Toxoplasma gondii*, serology

## Abstract

*Background and Objectives*: High rates of infection with *Toxoplasma gondii* were found in psychiatric patients globally. In this study, we assessed for the first time the prevalence of *T. gondii* infection in psychiatric patients and healthy individuals with no known psychiatric disorders in Western Romania. *Materials and Methods*: The presence of specific IgG anti-*T. gondii* antibodies was evaluated in 308 psychiatric patients and 296 control subjects using a chemiluminescence assay. *Results*: Overall, the seroprevalence of IgG antibodies was higher in psychiatric patients (67.86%; 209/308), compared with the control group (54.05%; 160/296) (*p* < 0.001). Our results revealed a significantly higher prevalence of *T. gondii* antibodies among patients with schizophrenia (69.77%), organic (personality and behaviour) disorders (76.74%), and mental disorders concerning alcohol abuse (84.62%), compared with the control group (*p* = 0.009, *p* = 0.005, *p* = 0.043, respectively). *Conclusions*: This study provides new and important information on the seroprevalence of *T. gondii* in Romanian psychiatric patients and may serve for further scientific research regarding the status of *T. gondii* infection in patients with psychiatric disorders.

## 1. Introduction

About one-third of the world’s population is infected with *Toxoplasma gondii*, the most common obligate intracellular protozoan parasite. *T. gondii* seroprevalence differs widely between countries [1,2,3]. Humans can be infected by ingestion or handling of undercooked or raw meat, ingestion of contaminated food or water [2,4], organ transplantation, via blood or leucocytes, or vertically from the acutely infected pregnant women to the foetus [2,5]. In immunocompetent subjects, the postnatally acquired *T. gondii* infection is generally considered clinically insignificant [3]. Under the pressure of the human’s immune response [2], *T. gondii* will transform into intracellular cysts (in the liver, muscle, and neuronal cells) and will persist for the host’s life [2,6]. It has been recently suggested that chronic infection with *T. gondii* in the brain correlates with changes in neuronal architecture, neurochemistry, and behaviour [7]. Several studies documented the connection between infection with *T. gondii* and certain mental health disorders such as schizophrenia [8], bipolar disease [9], suicide attempts [10], personality disorders [11] and episodes of self-directed violence [12].

In Western Romania, the seroprevalence of *T. gondii* antibodies in the general adult population is 64.8% [13], but limited data regarding the prevalence of *T. gondii* infection in Romanian psychiatric patients are currently available to the international medical community. Therefore, we evaluated the presence of specific IgG anti-*T. gondii* antibodies in psychiatric patients and control subjects. 

## 2. Materials and Methods

### 2.1. Study Design and Population

A case–control study was conducted between 1 July 2018 and 31 July 2019. The study group included 308 consecutive psychiatric patients who were admitted to the Psychiatric Clinic of County Emergency Hospital in Arad, Romania. Clinical diagnoses were established in accordance with the *Diagnostic and Statistical Manual of Mental Disorders*, fifth edition (DSM-V) [14]. Mental illnesses were further classified using the International Classification of Disease, 10th Revision (ICD-10) codes (https://www.icd10data.com/ICD10CM/Codes) (accessed on 29 December 2021).

The control group consisted of 296 consecutive volunteer blood donors, with no known psychiatric disorders, referred to the Regional Blood Transfusion Centre in Timisoara, Romania. All donors had to comply with the donation eligibility criteria set by the Romanian Ministry of Health [15]. 

This study was approved by the Ethics Committees of the Emergency County Hospital in Arad, Romania, and Victor Babes University Ethics Committee in Timisoara, Romania. All participants were aged over 18 years (with no upper age limit) and, after the study procedures and goals were carefully explained to them, they provided written informed consent. The sociodemographic characteristics (gender and age) were recorded for all subjects included in the study. The age given for each study participant is the age at which the serum sample was drawn.

### 2.2. Serologic Tests

Blood samples were collected at study enrolment, using the standard venepuncture method, into serum separation gel and clot activator vacuum tubes. The collected samples were then centrifuged at 4000× *g* for 10 min, in 10 to 30 min after collection. The obtained sera were moved into sterile centrifuge Eppendorf tubes and stored at −20 °C until tested.

*T. gondii* IgG antibodies were determined using chemiluminescence on Immulite 2000 analyser (Siemens Healthcare Diagnostics, Malvern, PA, USA). The test kits were used according to the manufacturer’s protocol (including quality controls), and interpretation of the results was based on the manufacturer’s criteria, as follows: <6.5 IU/mL, negative; ≥6.5 IU/mL to 7.99 IU/mL, equivocal; ≥8 IU/mL, positive. For the purpose of this study, equivocal results were considered negative. 

### 2.3. Statistical Analysis

Data were recorded in a Microsoft Excel database, version 2011 (Microsoft Corp., Redmond, WA, USA). Statistical analyses were conducted using the Epi Info statistical package 3.3.2 (Centres for Disease Control and Prevention, Atlanta, GA, USA). Means, standard deviations, and proportions are presented. Crude odds ratios (ORs) and their 95% confidence intervals (95% CIs) were calculated. To evaluate differences in *T. gondii* seroprevalence between groups, we used Mantel–Haenszel chi-square and two-tailed Fisher’s exact tests, as appropriate. We consider *p* < 0.05 statistically significant.

## 3. Results

The 308 psychiatric patients included in the study group were aged between 19 and 63 years (mean age = 45.64 ± 9.90 years), and 142 (46.10%) were males. The control group consisted of 296 subjects aged between 19 to 63 years (mean age = 45.29 ± 9.94 years), and 143 (48.31%) were males (Table 1). No statistically significant difference in age was found between cases and controls (*p* = 0.669).

The seroprevalence of *T. gondii* increased with age, in both psychiatric patient and blood donor groups (data not shown). However, the overall seroprevalence of *T. gondii* IgG antibodies was significantly higher in psychiatric patients (67.86% 209/308), compared with blood donors (54.05%, 160/296, OR:1.79; 95%CI: 1.29–2.50; *p* < 0.001) (Table 2).

Further analysis with stratification by sex showed a higher significant difference in *T. gondii* seroprevalence between the psychiatric female patients and female controls (73.49%, 122/166 vs. 54.25%, 83/153; OR: 2.33; 95%CI: 1.46–3.74; *p* < 0.001), but no significant difference was found when psychiatric male patients were compared with male controls (61.27%, 87/142 vs. 53.85%, 77/143; OR: 1.35; 95%CI: 0.85–2.17; *p* = 0.231) (Table 2).

The seroprevalences of *T. gondii* IgG antibodies in patients according to their psychiatric disorder are presented in Table 3. When compared with the control group, a significantly higher prevalence of *T. gondii* antibodies was found among patients with schizophrenia (69.77%; OR = 1.96; 95%CI: 1.17–3.28; *p* = 0.009), organic (personality and behaviour) disorders (76.74%; OR = 2.81; 95%CI: 1.33–5.90; *p* = 0.005), and mental disorders concerning alcohol abuse (84.62%; OR = 4.68; 95%CI: 1.02–21.46; *p* = 0.043) (Table 3).

## 4. Discussion

This is the first case–control study to evaluate the seroprevalence of *T. gondii* infection in Romanian psychiatric patients, compared with healthy blood donors. Blood donors were used as a control group due to the strict criteria [15]; they need to donate blood, which means their health conditions are as feasibly close as possible to the definition of the term ‘healthy’. Our results revealed a high prevalence of *T. gondii* IgG antibodies in patients diagnosed with schizophrenia, organic (personality and behaviour) disorders, and mental disorders concerning alcohol abuse, compared with controls. Similar to the results of other studies conducted in this region [4,13], the prevalence of *T. gondii* antibodies tended to increase with age in both groups: psychiatric patients and controls.

In immunocompetent individuals, *T. gondii* infection is generally asymptomatic. However, in recent years, a strong relationship has emerged between *T. gondii* infection and psychiatric disorders [16]. *T. gondii*, by direct stimulation of inflammatory cytokines in the central nervous system, can cause brain inflammation. It can also act on neurotransmitters (especially dopamine) that are involved in the development of psychosis and behavioural abnormalities [17]. Another hypothesis suggests that, in subjects infected with *T. gondii*, the onset of schizophrenia is triggered by an increased concentration of kynurenic acid (degradation product of tryptophan) which will inhibit glutamine and nicotine neurotransmitter receptors [18]. It has recently been suggested that chronic infection with *T. gondii* in the brain correlates with changes in neuronal architecture, neurochemistry, and behaviour [7].

The results of studies regarding the association between *T. gondii* infection and psychiatric disorders are controversial. Although this association has been confirmed by many studies [19,20,21,22,23,24,25], there are published data that suggest a lack of association [16,18,26,27,28,29].

In the present study, we found a 67.86% seroprevalence of *T. gondii* IgG antibodies among Romanian psychiatric patients, higher than the 54.7% prevalence recently reported in patients with psychiatric disorders diagnosed in Western Romania [30].

The potential association of *T. gondii* infection with schizophrenia found in our study has previously been demonstrated by many authors [16,30,31,32]. However, unlike other studies [33,34], we found no association between the seroprevalence of *T. gondii* and bipolar disorder. Consistent with our findings, Yolken et al. revealed that individuals diagnosed with a delusional disorder had significantly increased seroprevalence of *T. gondii* IgG antibodies, compared with controls, and did not differ significantly in individuals with bipolar disorder [29]. The concept that *T. gondii* seropositivity is lifelong has been called into question by some authors, who suggested that persistent exposure to the parasite is required for the maintenance of antibody levels [35]. Moreover, medications used to treat schizophrenia or bipolar disorder have anti-*Toxoplasma* activity in cell culture and can lead to the decline in *T. gondii* seropositivity over time [36].

Our results revealed no difference in the seroprevalence of *T. gondii* antibodies in patients diagnosed with depressive disorders, compared with blood donors, and are in contrast with the findings reported by Alvarado-Esquivel et al. [37]. Further studies should be conducted to assess the potential relationship of *T. gondii* with depressive disorders since recent data suggest that no association can be found between latent *T. gondii* infection and major depressive disorders [38]. It has been previously shown that *T. gondii* may cause depression and mood disorders by affecting serotonin and/or dopamine biosynthesis, the tryptophan metabolism, and the hypothalamic–pituitary–adrenal axis [39]. In the present study, a significant difference in *T. gondii* seroprevalence was found between patients diagnosed with mental disorders concerning alcohol abuse and healthy blood donors. Our results are consistent with those found by Samojłowicz et al. [40] and Estrada-Martinez et al. [41] but inconsistent with the results published by Suvisaari et al. [42], who found no association between *T. gondii* seropositivity and alcohol abuse.

*T. gondii* past infection was not found to be related to mental retardation in our study group, similar to results published by Ezatpour et al. [43].

In contrast with the results published by other authors [7,19,37], we found a higher seroprevalence of *T. gondii* antibodies in females with psychiatric disorders, compared with females from the control group. No questionnaire was used in this study to identify the risk factors associated with *T. gondii*, and therefore, we cannot explain the difference in seroprevalence. Further epidemiological surveys should be conducted to identify the potential risk factors for *T. gondii* infection in psychiatric patients.

## 5. Conclusions

Results of the present study indicate a significantly higher *T. gondii* IgG seroprevalence rate in psychiatric patients, compared with controls. The prevalence of *T. gondii* IgG antibodies was higher in patients diagnosed with schizophrenia, organic (personality and behaviour) disorders, and mental disorders concerning alcohol abuse. This study brings new and important data regarding the seroprevalence of *T. gondii* in patients with psychiatric diseases. Further epidemiological studies are needed to assess the risk factors for toxoplasmosis in psychiatric patients to better understand the potential relationship between *T. gondii* infection and psychiatric disorders.

## Figures and Tables

**Table 1:** Demographic characteristics of the study group (psychiatric patients) and the control group (blood donors) included in the study.

Characteristics	Study Group (*n* = 308, 100%)	Control Group (*n* = 296, 100%)
**Age** (Mean ± SD)	45.64 ± 9.90	45.29 ± 9.94
**Sex**		
Male	142 (46.10%)	143 (48.31%)
Female	166 (53.90%)	153 (51.69%)
**Age Groups** (years)		
19–29	24 (7.79%)	24 (8.11%)
30–39	53 (17.21%)	53 (17.91%)
40–49	96 (31.17%)	96 (32.43%)
50–59	128 (41.56%)	116 (39.19%)
≥60	7 (2.27%)	7 (2.36%)

**Table 2:** Seroprevalence of *T. gondii* IgG antibodies in the study and control groups, according to sex.

	Psychiatric Patients (Study Group)		Blood Donors (Control Group)		OR (95%CI)	*p* Value
	No. Positive (%)	No. Negative (%)	No. Positive (%)	No. Negative (%)		
Total	209 (67.86%)	99 (32.14%)	160 (54.05%)	136 (45.95%)	1.79 (1.29–2.50)	<0.001
**Sex**						
Male	87 (61.27%)	55 (38.73%)	77 (53.85%)	66 (46.15%)	1.35 (0.85–2.17)	0.231
Female	122 (73.49%)	44 (26.51%)	83 (54.25%)	70 (45.75%)	2.33 (1.46–3.74)	0.001

**Table 3:** Seroprevalence of *Toxoplasma gondii* IgG antibodies in psychiatric patients from Western Romania, according to diagnosis.

Diagnosis	ICD-10 Diagnosis	Total No. Tested	No. Tested Positive (%)	OR (95%CI)	*p* Value *
Schizophrenia	F20.9	86	60 (69.77%)	1.96 (1.17–3.28)	0.009
Dementia	F03.90	4	2 (50%)	0.85 (0.12–6.12)	1
Organic (personality and behaviour) Disorders	F07.8	43	33 (76.74%)	2.81 (1.33–5.90)	0.005
Bipolar disorders	F31.9	20	11 (55%)	1.03 (0.42–2.58)	1
Mental Disorder concerning alcohol abuse	F10.1	13	11 (84.62%)	4.68 (1.02–21.46)	0.043
Depressive disorder	F33.0	75	48 (64%)	1.51 (0.89–2.55)	0.152
Delusional disorder	F05.8	18	14 (77.78%)	2.98 (0.96–9.25)	0.054
Impulsive-control disorder	F63.9	11	7 (63.64%)	1.49 (0.43–5.19)	0.759
Mood disorder	F06.3	30	18 (60%)	1.28 (0.59–2.74)	0.569
Adjustment disorder	F43.2	2	0 (0%)	NA **	0.214
Mental retardation	F78.8	6	5 (83.33%)	4.25 (0.49–36.82)	0.226

* The seroprevalence of *T. gondii* IgG antibodies was compared with seroprevalence in control group. ** not applicable.

## Data Availability

Datasets used and/or analysed during the current study are available from the corresponding author on reasonable request.
